# IFN-CSP Inhibiting Hepatitis B Virus in HepG2.2.15 Cells Involves JAK-STAT Signal Pathway

**DOI:** 10.1155/2015/959684

**Published:** 2015-03-16

**Authors:** Xuemei Lu, Jie Wang, Xiaobao Jin, Yanting Huang, Wenting Zeng, Jiayong Zhu

**Affiliations:** ^1^School of Basic Courses, Guangdong Pharmaceutical University, Guangzhou Higher Education Mega Center, 280 Wai Huan Dong Road, Guangzhou 510006, China; ^2^Guangdong Provincial Key Laboratory of Pharmaceutical Bioactive Substances, Guangzhou Higher Education Mega Center, 280 Wai Huan Dong Road, Guangzhou 510006, China

## Abstract

Frequent and high-dose administration of interferon to patients with viral hepatitis results in various side effects. In our previous study, a novel liver-targeting interferon (IFN-CSP) combining *Plasmodium* region I peptide with IFN*α*2b was successfully designed and expressed in the *Escherichia coli* expression systems. This targeting would target the IFN*α*2b specifically to the liver, thus reducing the adverse events. In the present study, we further investigated the anti-HBV effects and molecular mechanisms of recombinant IFN-CSP in HepG2.2.15 cell line. Hepatitis B surface antigen (HBsAg) and HBe antigen (HBeAg) in the culture supernatants were analyzed by enzyme-linked immunosorbent assay (ELISA). HBV-DNA was measured by real-time quantitative PCR. HBV core protein was assayed by immunofluorescent and western blot analysis. The expressions of signal transducers and transactivator 1 (STAT1), STAT2, IFN regulatory factor 9 (IRF-9), and 2′-5′-oligoadenylate synthetase 1 (OAS1) were investigated by the reverse transcription PCR and western blot analysis. Results indicate IFN-CSP efficiently inhibited HBsAg and HBeAg secretion, HBV-DNA replication, and HBV core protein expression in HepG2.2.15 cells. The anti-HBV mechanisms involve activation of JAK-STAT signaling and increase of the anti-HBV protein OAS expression. IFN-CSP could be a good substitute for IFN*α*2b for anti-HBV treatment.

## 1. Introduction

Hepatitis B virus (HBV) is a DNA virus that spreads through infected blood or body secretions [1]. More than 400 million people are chronically infected with HBV worldwide [2, 3]. China has one of the largest HBV infected populations in the world [4]. HBV infection is directly related to the life expectancy due to acute, chronic hepatitis, cirrhosis and hepatocellular carcinoma (HCC) caused by HBV [5, 6]. Despite the availability of efficient vaccines, HBV infection remains a major global health concern.

The interferons (IFNs) are cytokines that exhibit antiviral, immunomodulatory, and antiproliferative activities [7–10]. The biomedical interest in interferons is linked to their therapeutic efficacy against viral infections and cancer [11]. However, a variety of shortcomings have limited the widespread use of interferons, including the inability to obtain clinically relevant concentrations* in vivo* and limited access of exogenous interferons to the site of infection [12]. Novel strategy to enhance the* in vivo* efficacy of IFN by targeting it to specific site might circumvent these problems. Incorporation of* Plasmodium* region I peptide was demonstrated to be a promising strategy for the development of liver-targeting drug [13, 14]. In our previous study, a novel liver-targeting fusion interferon (IFN-CSP) combining* Plasmodium* region I peptide with IFN *α*2b was successfully designed and expressed in the* Escherichia coli* expression systems [15]. Liver tissue binding analysis revealed IFN-CSP specific targeting to liver tissue [15]. However, the anti-HBV effect of IFN-CSP still needs to be further studied and its precise molecular mechanism has not been identified.

To evaluate the anti-HBV effects and molecular mechanisms of recombinant IFN-CSP* in vitro*, HepG2.2.15 cell was used as* in vitro* model. Hepatitis B surface antigen (HBsAg) and hepatitis B e antigen (HBeAg) in the culture supernatants were analyzed by enzyme-linked immunosorbent assay (ELISA). HBV-DNA was measured by real-time quantitative PCR. HBV core protein was assayed by immunofluorescent and western blot analysis. The expressions of signal transducers and transactivator 1 (STAT1), STAT2, IFN regulatory factor 9 (IRF9), and 2′-5′-oligoadenylate synthetase 1 (OAS1) were investigated by the reverse transcription-polymerase chain reaction (RT-PCR) and western blot analysis.

## 2. Materials and Methods

### 2.1. Cell Culture and Materials

HepG2.2.15 cell, which has been stably transformed with two copies of the HBV genome into human hepatoblastoma cell line HepG2, was used as* in vitro* model to evaluate the anti-HBV effect and mechanism of IFN-CSP. The HepG2.2.15 cells were grown in complete DMEM (Gibco-BRL, CA) containing 10% FBS (Hyclone, Thermo Fisher, PA), 380 micro·g/mL G418 antibiotic (Sigma, MO), 100 units/mL penicillin, and 100 mg/mL streptomycin in a humidified incubator with 5% CO_2_ at 37°C.

IFN*α*2b was purchased from Beijing Kaiyin Pharmaceutical Group (Beijing, China). IFN-CSP was expressed in* Escherichia coli*, with a specific activity of 1.2 × 10^8^ IU/mg protein at a purity of over 95% [15].

### 2.2. MTT Analysis for Cytotoxicity

HepG2.2.15 cells were seeded into 96-well plates (2 × 10^4^/well) with IFN*α*2b and various concentrations of IFN-CSP for 9 days. The culture medium was replaced every 3 days. The cells with media alone were used as controls. MTT solution (5 g/L) was added in the end of the incubation, and DMSO was added to solubilize the formazan. Optical density (OD) values were read at 570/630 nm. The survival ratio (%) was calculated using

(1) $$\begin{array}{c} \text{Survival}\;\;\text{rate}\left(\%\right)=\left(\frac{{\text{OD}}_{\text{sample}}}{{\text{OD}}_{\text{control}}}\right)\times 100\%. \end{array}$$

### 2.3. Antiviral Experiments

Antiviral experiments were performed as previously described [16]. Briefly, the HepG2.2.15 cells were placed in 24-well plates (2 × 10^5^/well) and incubated for 24 h. Then, the culture medium was replaced by fresh medium with IFN*α*2b (at final a concentration of 10^3^ IU/mL, *n* = 3) or IFN-CSP (at final concentrations of 10^1^, 10^2^, and 10^3^ IU/mL, *n* = 3) for 9 days. The medium alone was added to the control wells (*n* = 3). The culture medium was replaced every 3 days. The cells were harvested for HBV core protein assays. The supernatants were harvested for HBV antigens and HBV-DNA assays.

To investigate the molecular mechanism of the anti-HBV effect of IFN-CSP, confluent HepG2.2.15 cells were pretreated for 1 h with 10 *μ*M of AG 490, a JAK inhibitor, and subsequently stimulated cells with either IFN-CSP or IFN*α*2b for 3 days [17]. The supernatants were harvested for HBV antigens and HBV-DNA assays. The cells were harvested for signal transducers and transactivator 1 (STAT1), STAT2, IFN regulatory factor 9 (IRF-9), and 2′-5′-oligoadenylate synthetase 1 (OAS1) mRNA and OAS1 protein assays.

### 2.4. ELISA Analysis for HBsAg and HBeAg

HBsAg and HBeAg in the culture supernatants were determined by ELISA assay according to the manufacturer's recommendations (Shanghai Kehua Biotech Co., Ltd., China). The OD values at 450/630 nm were measured by ELISA reader (Bio-Rad, USA). The inhibitory rates were calculated using

(2) $$\begin{array}{c} \text{Inhibitory}\;\;\text{rate}\left(\%\right)=\left\{\frac{\left({\text{OD}}_{\text{control}}-{\text{OD}}_{\text{sample}}\right)}{{\text{OD}}_{\text{control}}}\right\}\times 100\%. \end{array}$$

### 2.5. Real-Time Quantitative PCR Analysis for HBV-DNA

HBV-DNA was isolated from the culture supernatants for the fluorescent quantification PCR. The load of HBV viral was determined using a HBV Fluorescent Quantitative PCR Detection kit (Da-An Gene Co., Ltd., China) according to the manufacturer's protocol. After initial denaturation at 93°C for 2 min, the PCR reaction was followed by 10 rounds of 93°C for 45 s and 55°C for 60 s and 30 rounds of 93°C for 30 s and 55°C for 45 s. HBV-DNA copies were quantified using a standard curve. The inhibition ratio (%) was calculated using

(3) Inhibitory  rate(%) =HBV-DNA  copiescontrol−HBV-DNA  copiessampleHBV-DNA  copiescontrol  ×100%.

### 2.6. Immunofluorescent and Western Blot Analysis for HBV Core Protein

HBV core protein was analyzed by both immunofluorescence and western blot analysis. For immunofluorescence staining and confocal microscope analysis, the treated cells were seeded on coverslip and fixed with 4% paraformaldehyde. After being permeabilized with 0.1% (vol/vol) triton X-100 for 30 min, immunofluorescent detection of HBV core protein was performed on HepG2.2.15 cells using rabbit polyclonal IgG anti-human HBcAg primary antibody (1 : 200; Abcam, England) and Alexa Fluor 488-conjugated goat anti-rabbit IgG (1 : 200; Beyotime, China). Finally, the cells were stained with 4′,6-diamidino-2-phenylindole (DAPI, Vector, CA) for nuclear indication. Images were captured using a confocal laser scanning microscope (TCS-NT, Leica Microsystems, Heidelberg, Germany).

For western blot analysis, the treated cells were lysed in lysis buffer and cell lysates were separated in an SDS 12% polyacrylamide gel. The separated proteins were transferred to PVDF membrane and detected using primary polyclonal anti-HBcAg antibody (1 : 200; Abcam, England), anti-actin monoclonal antibody (1 : 1000; Santa Cruz Biotechnology, USA), and peroxidase-conjugated secondary antibodies followed by ECL detection. Immunoreactive bands were digitized using a scanner and signal was quantified using Image-Pro Plus (IPP) software (Version 6.0, Media Cybernetics, USA).

### 2.7. Semiquantitative RT-PCR and Western Blot for JAK-STAT Signal Molecules

STAT1, STAT2, IRF9, and OAS1 mRNA were measured by semiquantitative RT-PCR. Total RNA was extracted with Trizol reagent (Invitrogen, USA) according to the manufacturer's instructions. The concentration and purity of total RNA were measured based on the* A*260 and* A*280 value and 1% agarose-formaldehyde gel electrophoresis. Synthesis of the complementary DNA was performed as described by the manufacturer using M-MLV reverse transcriptase (Promega, USA). PCR amplification was conducted on an ABI PRISM 7300 Sequence Detector System (Perkin-Elmer Applied Biosystems, CA) using PCR kit (Takara, China). The gene names, GenBank accession numbers, forward and reverse primer sequences, and product sizes are listed in Table 1. Each reaction was performed in three replicate samples. The *β*-actin gene was used as the standard. RT-PCR products were separated through 1.5% agarose gels. The signal was normalized by *β*-actin and analyzed by Quantity One software (Bio-Rad, USA).

OAS1 protein was measured and analyzed by a standard western blot procedure [18] using primary polyclonal anti-OAS1 antibody (1 : 200; Santa Cruz Biotechnology, USA), primary monoclonal anti-actin antibody, and peroxidase-conjugated secondary antibodies. The image was digitized using a scanner and signal was quantified using IPP software.

### 2.8. Statistical Analysis

All data were expressed as the mean ± standard error of the mean (SEM). The homogeneity of variances was checked with Levene's test, and differences were analyzed by One-Way ANOVA using SPSS (version 13.0 for Windows) statistical software. *P* < 0.05 is considered statistically significant.

## 3. Results

### 3.1. Effect of IFN-CSP on HBV Antigens Secretion and HBV-DNA Replication

**Figure 1:**
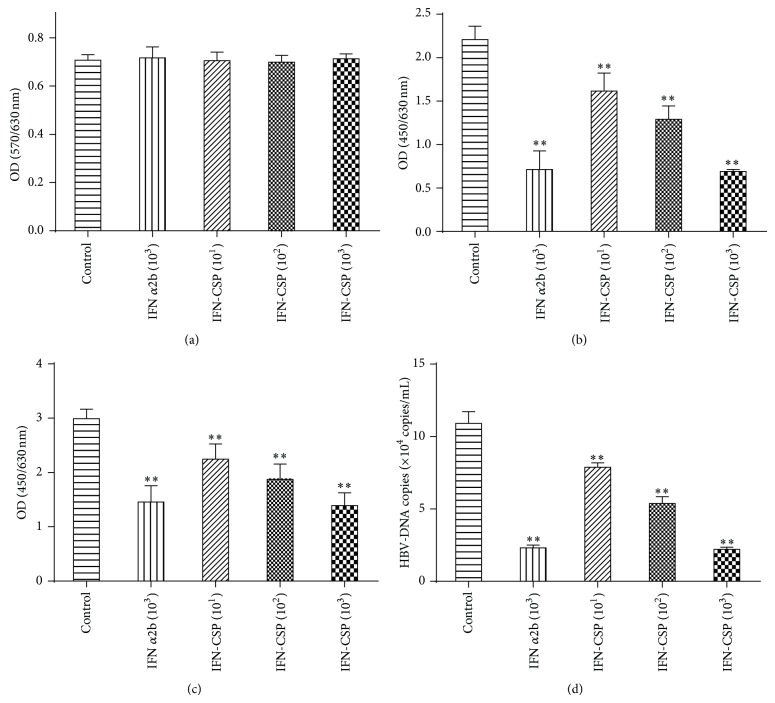
IFN-CSP treatment suppressed HBV antigens secretion and HBV-DNA replication in HepG2.2.15 cells. HepG2.2.15 cells were cultured in the presence of IFN*α*2b and IFN-CSP at various concentrations for 9 days and the cell survival rates were measured by MTT method (a). Hepatitis B surface antigen (HBsAg; b) and hepatitis B e antigen (HBeAg; c) in the culture supernatants were analyzed by enzyme-linked immunosorbent assay (ELISA). HBV-DNA (d) in the culture supernatants was measured by real-time quantitative PCR. Data represent the mean ± SEM of three experiments. ^**^ *P* < 0.01 as compared with the no-drug control group.

The HBsAg and HBeAg in the supernatant of HepG2.2.15 cell culture system were determined by ELISA diagnostic kit. Treatment with IFN-CSP resulted in a significant reduction of HBsAg (Figure 1(b)) and HBeAg (Figure 1(c)) secretion in a dose-dependent manner. The secretions of HBsAg were significantly reduced, with inhibition ratios of 26.86, 38.92, and 66.01%, respectively. The inhibition ratio of IFN*α*2b on HBsAg was 64.83%. For HBeAg secretions, the inhibition ratios of IFN-CSP were 24.95, 37.40, and 53.53%, respectively. The inhibition ratio of IFN*α*2b on HBeAg was 51.30%. To further confirm the anti-HBV activity of IFN-CSP, the HBV-DNA levels in the culture supernatant were measured. The results revealed that the abundance of HBV-DNA (Figure 1(d)) released into the culture medium was significantly decreased in a dose-dependently manner, with inhibition ratios of 27.82, 50.69, and 79.77%, respectively. The inhibition ratio of IFN*α*2b on HBV-DNA was 78.91%. IFN*α*2b and IFN-CSP at the concentration of tested (10^3^ IU/mL) showed no cytotoxicity on the HepG2.2.15 cells, as analyzed by the MTT assay (Figure 1(a)).

### 3.2. Inhibit the Expression of HBV Core Protein in HepG2.2.15 Cells by IFN-CSP Treatment

**Figure 2:**
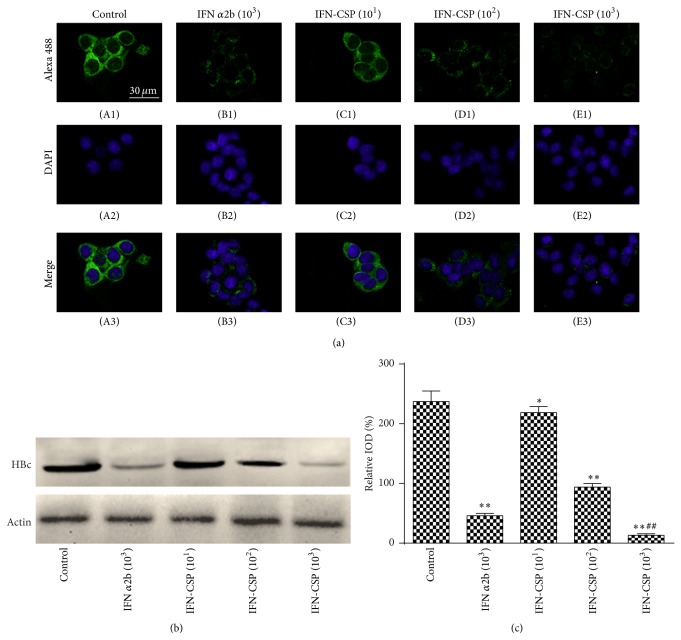
Analysis of HBV core protein in HepG2.2.15 cells treated with IFN-CSP for 9 days. (a) HepG2.2.15 cells stained by immunofluorescent staining with anti-HBcAg antibody and scanned by confocal microscope. Representative photographs are presented. Green stained with HBV core protein. Blue nuclear stained with DAPI. Calibration bar = 30 *μ*m for all images. (b) The HBV core protein was also measured by western blot analysis. (c) Optical densities of the core protein were analyzed using Image-Pro Plus (IPP) software. The data are the mean ± SEM (*n* = 3). ^*^ *P* < 0.05 versus untreated controls, ^**^ *P* < 0.01 versus untreated controls, and ^##^ *P* < 0.01 versus IFN *α*2b controls.

The expression level of HBV core protein in HepG2.2.15 cells was assayed by immunofluorescent staining and western blot analysis. The photographs scanned by confocal microscope (Figure 2(a)) showed that the signal of HBV core protein is obviously decreased in the cytoplasm of HepG2.2.15 cells treated with IFN-CSP in a dose-dependent manner when compared with the control cells. IFN*α*2b control also suppress the expression of HBV core protein in HepG2.2.15 cells, but the suppress degree less than the same dose of IFN-CSP. The result of western blot (Figures 2(b) and 2(c)) was consistent with the immunofluorescent staining.

### 3.3. IFN-CSP Upregulates STAT1, STAT2, IRF-9, OAS1, mRNA Expression and OAS1 Protein Expression in HepG2.2.15 Cells

**Figure 3:**
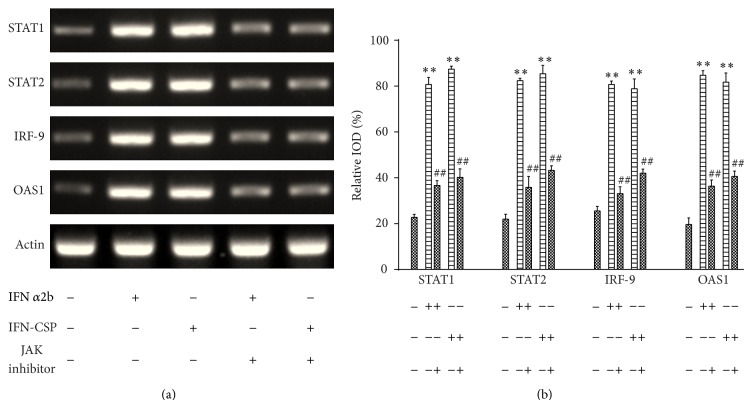
Stimulation of IFN-CSP on the mRNA expression of STAT1, STAT2, IRF-9, OAS1, and blockade by JAK inhibitor. Confluent HepG2.2.15 cells were pretreated for 1 h with JAK inhibitor or not and then incubated with either IFN-CSP or IFN*α*2b for 3 days. The levels of STAT1, STAT2, IRF-9, and OAS1 mRNA were assayed by RT-PCR (a), and the *β*-actin gene was used as an internal control. The corresponding relative integrated optical densities (IOD) of the gene band were analyzed using Image-Pro Plus (IPP) software (b). Data are the mean ± SEM of three experiments. ^**^ *P* < 0.01 versus untreated controls and ^##^ *P* < 0.01 versus no JAK inhibitor controls.

**Figure 4:**
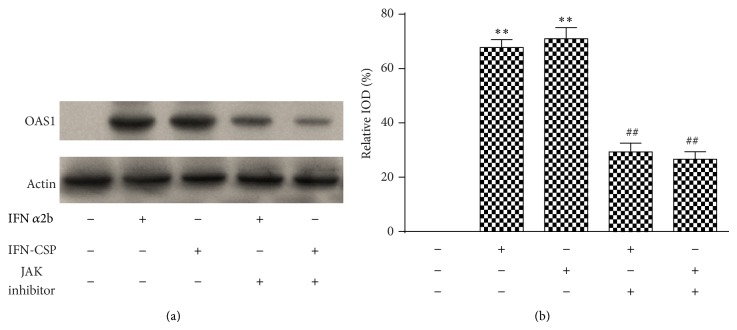
Stimulation of IFN-CSP on the protein expression of OAS1 and blockade by JAK inhibitor. Confluent HepG2.2.15 cells were pretreated for 1 h with JAK inhibitor or not and then incubated with either IFN-CSP or IFN*α*2b for 3 days. The levels of OAS1 protein in HepG2.2.15 cells were assayed by western blot (a) and the *β*-actin gene was used as an internal control. The corresponding relative integrated optical densities (IOD) of the gene band were analyzed using Image-Pro Plus (IPP) software (b). Data are the mean ± SEM of three experiments. ^**^ *P* < 0.01 versus untreated controls, ^##^ *P* < 0.01 versus no JAK inhibitor controls.

The ability of type I IFN to activate the JAK-STAT pathway prompted us to investigate how the signaling pathway affected the gene expression induced by IFN-CSP. Therefore, we treated the HepG2.2.15 cells with either IFN-CSP or IFN*α*2b. RNA and protein were harvested for measurement of the pivotal factors in the JAK-STAT pathway. As shown in Figure 1, a clear increase in STAT1, STAT2, IRF-9, and OAS1 mRNA expressions was seen after stimulation with both IFN-CSP and IFN*α*2b (Figure 3). We also observed a significant increase in OAS1 protein expression after treatment with both IFN-CSP and IFN*α*2b (Figure 4).

### 3.4. Blockade of JAK Activity Inhibits IFN-CSP Induced JAK-STAT Signaling

The possible involvement of JAK-STAT signal pathway in IFN-CSP induced gene expression in HepG2.2.15 cells was further studied by using AG 490 (Beyotime, China), a JAK inhibitor. Pretreatment of the cells for 1 h with AG 490 (10 *μ*M) significantly attenuated the gene expression of STAT1, STAT2, IRF-9, and OAS1 (Figure 3) and the protein expression of OAS1 (Figure 4) stimulation by IFN-CSP.

**Figure 5:**
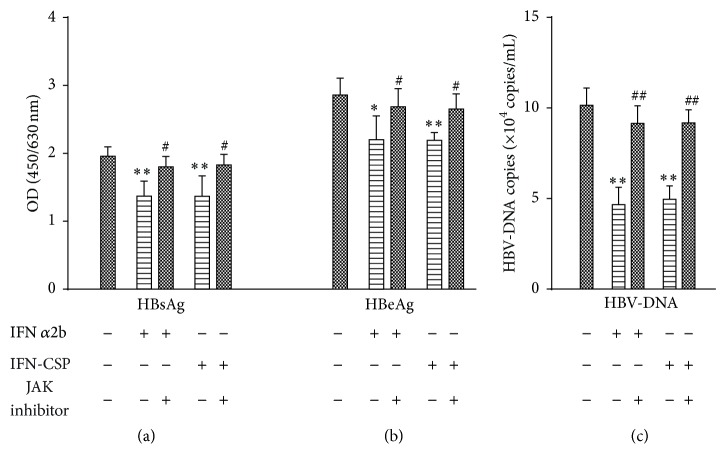
Blockade of the anti-HBV effect of IFN-CSP by JAK inhibitors. Confluent HepG2.2.15 cells were pretreated for 1 h with JAK inhibitor or not and then incubated with either IFN-CSP or IFN*α*2b for 3 days. Hepatitis B surface antigen (HBsAg; a) and hepatitis B e antigen (HBeAg; b) in the culture supernatants were analyzed by enzyme-linked immunosorbent assay (ELISA). HBV-DNA (c) in the culture supernatants was measured by real-time quantitative PCR. Data represent the mean ± SEM of three experiments. ^*^ *P* < 0.05 versus untreated controls ^**^ *P* < 0.01 versus untreated controls, ^#^ *P* < 0.05 versus no JAK inhibitor controls, ^##^ *P* < 0.01 versus no JAK inhibitor controls.

To assess whether AG 490-induced inhibition of JAK-STAT signaling translated into impaired anti-HBV effect of IFN-CSP, HBV antigens secretion and HBV-DNA replication in HepG2.2.15 cells were measured. After treatment of HepG2.2.15 cells for 3 days, the inhibition ratio of IFN-CSP on HBsAg, HBeAg, and HBV-DNA was 30.18%, 23.35%, and 51.14%, respectively. When HepG2.2.15 cells were pretreated with AG 490, the inhibition ratio (6.61%, 6.19%, and 9.68%) significantly decreased (Figure 5).

## 4. Discussion

The present study demonstrates clearly that the novel liver targeting interferon IFN-CSP efficiently inhibited HBsAg and HBeAg secretion, HBV-DNA replication, and HBV core protein expression in HepG2.2.15 cells. The results of our study also showed that IFN-CSP can act on HepG2.2.15 cells to induce the antiviral enzyme OAS1 through activating JAK-STAT signaling pathway. In our previous report, the IFN-CSP has been demonstrated that has liver-targeting potentiality [15]. Thus, it appears reasonable to speculate that IFN-CSP may accumulate in the liver tissue, improving the safety and efficacy of the therapeutic in comparison with free IFN *α*2b* in vivo*.

HBV marker is an indispensable tool for the evaluation of HBV infection, hepatitis, and cirrhosis caused by HBV. Of the many different HBV markers used in clinical settings, HBsAg and HBeAg are effective serum markers for diagnosis of HBV infection and predicting flare-ups during therapy and determining when to conclude treatment [19]. The technique for measuring HBsAg and HBeAg levels is enzyme-linked immunosorbent assay. In the present study, the HepG2.2.15 cell line, containing multiple copies of the HBV (ayw subtype) genome that can stably secret HBV particles to supernatant, was used as* in vitro* model for identifying the secretion of HBV antigens [20–22]. The ELISA results showed that IFN-CSP significantly reduce the levels of HBsAg and HBeAg in the supernatant of HepG2.2.15 cells.

HBV-DNA quantification is for diagnosis of breakthrough hepatitis via HBV mutation, evaluation of therapeutic effects, and assessment of liver disease [23]. High HBV-DNA levels also indicate a high risk of cancer. Since real-time PCR assay offers a wider measurement range and greater sensitivity, it is now recommended for HBV-DNA quantification in clinical settings. Significant depression in extracellular HBV-DNA levels was observed in IFN-CSP treated HepG2.2.15 cells in the present study. Consistent with the inhibitory effects on HBsAg, HBeAg and HBV-DNA, the results of immunofluorescent staining and western blot assay showed that IFN-CSP obviously decreased the level of cytoplasmic HBV core protein in HepG2.2.15 cells and a slightly better inhibit ability than native IFN *α*2b (Figure 2).

Moreover, it is critical to use concentrations that are not overtly cytotoxic since any impairment to cell functions would affect virus replication [24]. Our results showed that administration of the indicated concentration of IFN-CSP for 9 days did not induce cell death, indicating IFN-CSP achieved the anti-HBV effect without cytotoxicity on HepG2.2.15 cells.

The antiviral activities were dependent on IFN regulation of different signaling pathways. IFN stimulates a series of signal transduction events through binding of interferon to cell surface receptor. Among these pathways, the JAK-STAT signaling appeared to play a crucial role. The heteromeric IFN-stimulated gene factor 3 (ISGF3) complex, composed of STAT1, STAT2, and IRF-9, is a pivotal transcription factor in the JAK-STAT pathway [25]. In the study reported here, STAT1, STAT2, and IRF-9 mRNA levels in HepG2.2.15 cells treated with IFN-CSP 3 days were increased, consistent with the IFN*α*2b controls. This indicates IFN-CSP could also stimulate JAK-STAT signal transduction. OAS is considered to be the most sensitive biomarker of the effectiveness of IFN exposure, and it plays a critical role in mediating antiviral actions of interferon. In our experiments, a robust increase of the expressions of OAS1 mRNA and OAS1 protein by administration of IFN-CSP was similarly observed in HepG2.2.15 cells. The above information was further supported by the ability of a JAK inhibitor to attenuate the expression of STAT1, STAT2, IRF-9, and OAS1 induced by IFN-CSP. Moreover, impairment of JAK activity by the JAK inhibitor blockaded IFN-CSP induced anti-HBV effect in HepG2.2.15 cells. Thus, one of the possible mechanisms of the anti-HBV activity of IFN-CSP is activation of JAK-STAT signal transduction, followed by an increase in OAS.

In conclusion, we demonstrated that IFN-CSP efficiently inhibited HBsAg and HBeAg secretion, HBV-DNA replication, and HBV core protein expression in HepG2.2.15 cells. The anti-HBV mechanisms involve activation of JAK-STAT signaling and increase of the anti-HBV protein OAS expression. IFN-CSP could be a good substitute for IFN*α*2b for anti-HBV treatment.

## Figures and Tables

**Table 1:** Primer sequence for the semi-quantitative reverse transcription PCR used in this study.

Primer	GenBank accession number	Sequence (5′-3′)	RT-PCR product size
STAT1-Sense	NM_007315.3	TGGTGAAATTGCAAGAGCTG	214 bp
-Anti		GTTCTGGTGCCAGCATTTTT	

STAT2-Sense	NM_005419.3	CGACCAGAGCCATTGGAGGGCG	373 bp
-Anti		TCATCTCAGCCAACTGGGTAGG	

IRF-9-Sense	NM_006084.4	TGGCATCAGGCAGGGCACGCTG	409 bp
-Anti		GAACTGTGCTGTCGCTTTGATGG	

OAS1-Sense	NM_016816.2	AGGTGGTAAAGGGTGGCT	472 bp
-Anti		TGCTTGACTAGGCGGATG	

Actin-Sense	NM_001101	GGCATCGTGATGGACTCCG	613 bp
-Anti		GCTGGAAGGTGGACAGCGA	
